# Sample size determination through power simulation; practical lessons from a stepped wedge cluster randomised trial (SW CRT)

**DOI:** 10.1186/1745-6215-12-S1-A26

**Published:** 2011-12-13

**Authors:** Munyaradzi Dimairo, Mike Bradburn, Stephen J Walters

**Affiliations:** 1 CTRU, ScHARR, University of Sheffield, Sheffield, S1 4DA, UK; 2 MSG, ScHARR, University of Sheffield, Sheffield, S1 4DA, UK; 3 NIHR RDS for York & Humber, Sheffield, S1 4DA, UK

## Objectives

To describe the design of a stepped wedge randomised control trial (SW RCT) and demonstrate sample size estimation (total number of clusters and time periods) through power simulation when standard formulae are unavailable. 

## Methods

We describe the robustness of a stepped wedged design compared to other designs with respect to the referenced trial. We highlight difficulties faced during sample size estimation for a stepped wedge design in the presence of uncertainty of parameters and design constraints, and demonstrate how assessing the sensitivity can be achieved through power simulation.

## Results

Assuming the statistical power needs to be 80% or higher, a total of 6400 simulations will estimate the power to a standard error of 0.5%, In the present study, a total of 24 clusters and 5 time periods were required to give a study power of approximately 85% assuming a type I error rate of 5% and a 20% [RR=1.20] increase in lung cancer diagnosis rates attributable to the intervention within 6 months. Hence, 6 clusters will receive the intervention per each time period in a delayed intervention fashion and all clusters will act as controls during the first time period. Study power was insensitive to distributional assumptions under consideration in this case. 

## Conclusions

The stepped wedge CRT design is robust, flexible, offers a more practical alternative in evaluating intervention in routine practice than other designs and provides an opportunity to model time trends. Despite this, its implementation in routine practice is being hampered by complex statistical issues including sample size estimation. However, we have demonstrated that sample size calculation through power simulation is a simple and efficient approach when the use of routine formulae is impossible. Moreover, it is straightforward to assess sensitivity to issues such as heterogeneity across clusters, including the impact of design constraints such as centre or learning effects. We propose simulation-based power calculations should be considered routine practice in such circumstances. Finally, using a computer is easier than finding a book with the right formula.

